# Pathogenicity of *Aeromonas veronii* Isolated from Diseased *Macrobrachium rosenbergii* and Host Immune-Related Gene Expression Profiles

**DOI:** 10.3390/microorganisms12040694

**Published:** 2024-03-29

**Authors:** Xiaojian Gao, Zhen Chen, Zirui Zhang, Qieqi Qian, Anting Chen, Lijie Qin, Xinzhe Tang, Qun Jiang, Xiaojun Zhang

**Affiliations:** College of Animal Science and Technology, Yangzhou University, Yangzhou 225009, China; gaoxj336@163.com (X.G.); chenz_1103@163.com (Z.C.); zzr18762315883@163.com (Z.Z.); qieqi2000810@163.com (Q.Q.); chenanting2002@163.com (A.C.); 19352708430@163.com (L.Q.); txz001009@163.com (X.T.); jiangqun1013@163.com (Q.J.)

**Keywords:** *Macrobrachium rosenbergii*, *Aeromonas veronii*, pathogenicity, histopathology, immune-related genes

## Abstract

*Aeromonas veronii* is widespread in aquatic environments and is responsible for infecting various aquatic animals. In this study, a dominant strain was isolated from the hepatopancreas of diseased *Macrobrachium rosenbergii* and was named JDM1-1. According to its morphological, physiological, and biochemical characteristics and molecular identification, isolate JDM1-1 was identified as *A. veronii*. The results of artificial challenge showed isolate JDM1-1 had high pathogenicity to *M. rosenbergii* with an LD_50_ value of 8.35 × 10^5^ CFU/mL during the challenge test. Histopathological analysis revealed severe damage in the hepatopancreas and gills of the diseased prawns, characterized by the enlargement of the hepatic tubule lumen and gaps between the tubules as well as clubbing and degeneration observed at the distal end of the gill filament. Eight virulence-related genes, namely *aer*, *ompA*, *lip*, *tapA*, *hlyA*, *flgA*, *flgM*, and *flgN*, were screened by PCR assay. In addition, virulence factor detection showed that the JDM1-1 isolate produced lipase, lecithinase, gelatinase, and hemolysin. Furthermore, the mRNA expression profiles of immune-related genes of *M. rosenbergii* following *A. veronii* infection, including *ALF1*, *ALF2*, *Crustin*, *C-lectin,* and *Lysozyme,* were assessed, and the results revealed a significant upregulation in the hepatopancreas and intestines at different hours post infection. This study demonstrates that *A. veronii* is a causative agent associated with massive die-offs of *M. rosenbergii* and contributes valuable insights into the pathogenesis and host defense mechanisms of *A. veronii* invasion.

## 1. Introduction

China has become the world’s largest producer of *Macrobrachium rosenbergii*, a giant freshwater prawn, with an increase from 136,415 tons in 2000 to 177,836 tons in 2022 according to the China Fishery Statistical Yearbook [1]. *M. rosenbergii* is one of the most economically cultured freshwater species and is primarily found in tropical and sub-tropical areas [2]. With the rapid expansion of *M. rosenbergii* aquaculture in China, there has been a concurrent increase in the occurrence of diseases. In recent years, numerous pathogens have been identified as major contributors to high mortality rates in *M. rosenbergii* aquaculture.

Multiple pathogens have been implicated in causing mass mortality events in *M. rosenbergii*. Among these, *M. rosenbergii* nodavirus (*Mr*NV) [3], Decapod iridovirus Virus 1 (DIV1) [4], extra small virus (XSV) [5], Macrobrachium hepatopancreas parvovirus (MHPV) [6], white spot syndrome virus (WSSV) [7], and infectious hypodermal and hematopoietic necrosis virus (IHHNV) [8] have shown significant impact. Additionally, there has been an increasing frequency of outbreaks associated with bacterial pathogens, including *Vibrio parahaemolyticus* [9], *Vibrio alginolyticus*, *Vibrio vulnificus* [10], *Aeromonas dhakensis* [11], *Aeromonas* veronii, *Aeromonas* caviae [12], and *Lactococcus garvieae* [13]. Since July 2020, outbreaks of mass mortalities of *M. rosenbergii* have occurred in some farms in Jiangdu and Gaoyou Counties of Yangzhou City, Jiangsu Province, and recurring disease outbreaks have led to substantial economic losses in *M. rosenbergii* aquaculture. In our study, *A. veronii* was isolated from diseased prawns and was identified as the main pathogen.

*A. veronii* is a widely distributed bacterium that is commonly found in freshwater and estuary environments. This opportunistic pathogen has been identified as a causative agent in diseases affecting various organisms, including fish, terrestrial animals, and even humans [14]. It can cause epizootic ulcerative syndrome, fin rot, hemorrhagic septicemia, exophthalmia, and abdominal swelling in farmed fish species, including *Ctenopharyngodon idellas*, *Ictalurus punctatus*, and *Oreochromis niloticus* [15,16,17], and can cause carapace ulcers and red gill disease in *Litopenaeus vannamei* and *Macrobrachium nipponense* [11,18].

In this study, we investigated the epidemic status of diseased *M. rosenbergii* with mass mortality and isolated a pathogenic strain of *A. veronii* from the diseased prawns. Subsequently, we investigated the pathogenicity of *A. veronii* to *M. rosenbergii* by artificial challenge tests, histopathology analysis, and the detection of virulence-related genes and virulence factors of the bacteria. Moreover, changes in immune-related gene expression profiles of *M. rosenbergii* at different times post infection with *A. veronii* were analyzed by quantitative real-time PCR (qRT-PCR) to examine the immune response. This study presents key findings regarding the pathogenicity of *A. veronii* in *M. rosenbergii* and provides novel insights into the immune responses triggered by *A. veronii* infection in *M. rosenbergii*. 

## 2. Materials and Methods

### 2.1. Clinical Signs and Bacterial Isolation

Since July of 2020, outbreak of mass mortalities of *M. rosenbergii* occurred in some aquaculture farms situated in Jiangdu and Gaoyou Counties, Jiangsu Province, with a mortality rate exceeding 30% based on epidemiological investigation. The disease primarily affected adult prawns measuring over 6 cm in length. Diseased adult prawns exhibited symptoms such as reduced swimming ability, weakness, poor appetite, pale hepatopancreas, soft shells, and empty intestines. Infected *M. rosenbergii* were randomly selected from the farms and then disinfected with 75% ethyl alcohol. The tissues were sampled and inoculated on LB agar plates and incubated at 28 °C for 24 h. After incubation, dominant colonies were selected based on dominance and definite colony morphology and were re-streaked on LB agar plates in triplicate to obtain pure cultures. The JDM1-1 strain was then preserved in LB liquid medium supplemented with 30% (*v*/*v*) sterile glycerol at −40 °C.

### 2.2. Experimental Infection

Healthy *M. rosenbergii* (mean weight: 7.22 ± 0.46 g) were obtained from a farm in Yangzhou, Jiangsu Province, and confirmed to be free of pathogens based on the examination of bacteria and viruses. Prior to the experiment, the prawns were randomly subjected to bacterial isolation and the presence of DIV1, HPV, IHHNV, MBV, YHV, WSSV and TSV was determined by PCR assay using specific primers to confirm the health of *M. rosenbergii*. Animal treatments were strictly in accordance with the guidelines of the Animal Experiment Ethics Committee of Yangzhou University. The prawns were acclimated at 25 ± 1 °C for 7 d and fed a commercial feed (Jiangsu Fuyuda Food Products Co., Ltd., Yangzhou, China) twice daily. Then, the prawns were divided into five infected groups and one control group (30 prawns per group). Each tank measured 60 cm × 40 cm × 40 cm and was equipped with a 50 L flowthrough water at 25 ± 0.5 °C. The water was aerated to maintain a dissolved oxygen level of 5.0 mg/L detected by a dissolved oxygen meter (Shanghai lNESA Scientific Instruments Co., Ltd., Shanghai, China). For the bacterial challenge assay, strain JDM1-1 was incubated in LB liquid medium at 28 °C for 24 h, enumerated by a 10-fold serial dilution in sterile PBS and spread on LB nutrient agar plates. The experimental groups were intramuscularly injected with 50 μL of different concentrations of the JDM1-1 strain (1.8 × 10^4^, 1.8 × 10^5^, 1.8 × 10^6^, 1.8 × 10^7^, and 1.8 × 10^8^ CFU/mL per prawn). The control group was similarly injected with 50 μL sterile PBS (0.01 M, pH 7.4). The mortalities and clinical signs of infected prawns were observed and monitored for 7 days. Bacteria from the hepatopancreas of experimentally infected prawns were re-isolated to check whether death had been caused by the injected bacterium. Moreover, the median lethal dose (LD_50_) was calculated by assessing the cumulative experimental mortality using the Behreans and Karber method [19]. 

### 2.3. Histological Analysis

Tissues from the hepatopancreas and gills were collected from infected and control prawns. These tissues were preserved using Bouin’s solution, dewatered using different concentrations of ethanol, and finally embedded in paraffin. Serial sections of paraffin-embedded tissues of 5 μm in thickness were sectioned by a microtome, stained with hematoxylin and eosin (H&E), and observed under a light microscope for histopathological analysis.

### 2.4. Bacterial Identification

The morphological characteristics of isolate JDM1-1 were examined using a transmission electron microscope (TEM). Briefly, the centrifugation of the JDM1-1 isolate was performed at 5000× *g* for 20 min at a temperature of 4 °C. The collected cells underwent three rounds of washing with sterilized PBS (0.01 M, pH 7.4) and were then fixed by adding 2.5% glutaraldehyde solution (1 mL) and stored at 4 °C for 2 h. Subsequently, they were treated with 1% osmic acid for 30 min, followed by three consecutive 10 min washes with phosphate buffer (pH 7.4). Ethanol dehydration and epoxy resin injection into the fixed samples were performed. Ultrathin sections were prepared and stained by applying a mixture of uranyl acetate and lead citrate. Ultimately, three samples were examined utilizing a Zeiss EM10 transmission electron microscope, and magnification was set to 5000×.

Commercial biochemical identification tubes (Hangzhou Binhe Microorganism Reagent Co., Ltd., Hangzhou, China) were used for the analysis of biochemical characteristics, including oxidase, motility, v-p test, o-f test, lactose, maltose, mannitol, mannose, sorbitol, inositol, salicin, H_2_S production, α-methyl-d-glucoside, tartrate utilization, nitrate reduction, citrate utilization, mucate utilization, and malonate utilization. The results were observed following incubation at 28 °C for 48 h and were compared with Bergey’s Manual of Systematic Bacteriology [20]. Bacteria DNA extraction kits (TransGen Biotech Co., Ltd., Beijing, China) were utilized to extract the total genomic DNA of JDM1-1 for molecular identification. Afterwards, the extracted DNA was employed as a template for PCR amplification. The primers and the protocol used to analyze the 16S rRNA and the *gyrB* partial gene of the JDM1-1 isolate were established by Zhang et al. [21]. Sequence analysis was performed using the Basic Local Alignment Search Tool (BLAST) to determine homology, and phylogenetic trees of the 16S rRNA and *gyrB* sequences were created by MEGA 7.0. using the neighbor-joining method. The reliability of the neighbor-joining tree was estimated by bootstrap analysis with 1000 replicates.

### 2.5. Determination of Extracellular Enzymes and Hemolysin

For the gelatinase test, LB nutritional agar was supplemented with 1% gelatin. For the lipase test, 1% Tween-80 was utilized in the agar. For the caseinase test, 2.5% skim milk was added to the agar. The diastase test involved the addition of 2% starch to the agar. The urease test was conducted using 2% urea in the agar. For the lecithinase test, 10% egg yolk was utilized in the agar. Finally, β-hemolytic activity was assessed by incubating the nutrient agar with 7% rabbit erythrocytes. Plates were incubated at 28 °C for 24 to 48 h. Lytic halos visible around colonies were taken as an indication of activity in the assays.

### 2.6. Virulence-Related Gene Detection

Eight virulence-related genes—hemolysin (*hlyA*), outer membrane protein (*OmpA*), type IV pilus (*tapA*), lipoidase (*lip*), aerolysin (*aer*), and flagellin (*flgA*, *flgM*, *flgN*)—were detected based on PCR, and the primers used are listed in Table 1. PCR amplification and the related protocol were conducted following the methodology described by Chen et al. [22]. Using 1% agarose gel, the amplified PCR product was subjected to electrophoresis and subsequently analyzed.

### 2.7. Detection of Immune-Related Gene Expression after Infection Using qRT-PCR

For immune challenge assays, a total of 300 prawns were divided equally into a challenge and a control group. In the challenge group, the prawns were intramuscularly injected with 50 μL of isolate JDM1-1 (10^5^ CFU/mL). In the control group, the prawns were administered an intramuscular injection of 50 μL sterile saline. The hepatopancreas and intestines of 3 individuals from each group were sampled at 0, 6, 12, 24, and 48 h after injection and immediately frozen in liquid nitrogen and stored at −80 °C until use.

Then, total RNA was extracted from the tissue samples via TRIzol reagent (Tiangen Biotech Co., Ltd., Beijing, China) and 1 μg of RNA was reverse-transcribed to cDNA using TransScript One-Step gDNA Removal and cDNA Synthesis Supermix (Vazyme Biotech Co., Ltd., Nanjing, China). qRT-PCR was employed to assess the expression levels of immune-related genes such as anti-lipopolysaccharide factors (*ALF1*, *ALF2*, *ALF3*), *Crustin*, *Hemocyanin,* and *Lysozyme*. The primer sequences used are shown in Table 2, with 18S rRNA serving as the internal reference gene. Real-time quantitative PCR reactions and amplification protocols were conducted using ChamQ Universal SYBR Green RT-qPCR Master Mix (Vazyme Biotech Co., Ltd., Nanjing, China), and the levels of gene expression were determined by the 2^−ΔΔCT^ method. Statistical significance was assessed by one-way analysis of variance (ANOVA) with SPSS software version 17.0; the results are expressed as means ± standard deviation (SD) and differences were considered significant at *p* < 0.05.

## 3. Results

### 3.1. Pathogenicity of Isolate JDM1-1 

The mortality levels of *M. rosenbergii* infected with the JDM1-1 isolate are summarized in Table 3. All *M. rosenbergii* specimens infected with JDM1-1 at a bacterial concentration of 1.8 × 10^8^ CFU/mL were dead 7 days post infection. Mortality rates of 96.67%, 56.67%, 16.67%, and 13.33% were observed in *M. rosenbergii* infected with concentrations of 1.8 × 10^7^, 1.8 × 10^6^, 1.8 × 10^5^, and 1.8 × 10^4^ CFU/mL of JDM1-1, respectively, within a 7-day period. LD_50_ was calculated as 8.35 × 10^5^ CFU/mL using the Behreans and Karber method [19]. Furthermore, the symptoms of the artificially infected prawns were reduced swimming ability, weakness, poor appetite, and empty intestines. Additionally, bacterial isolates were re-isolated from the infected prawns; they exhibited the same morphological and biochemical features as isolate JDM1-1.

### 3.2. Histopathological Changes

**Figure 1:**
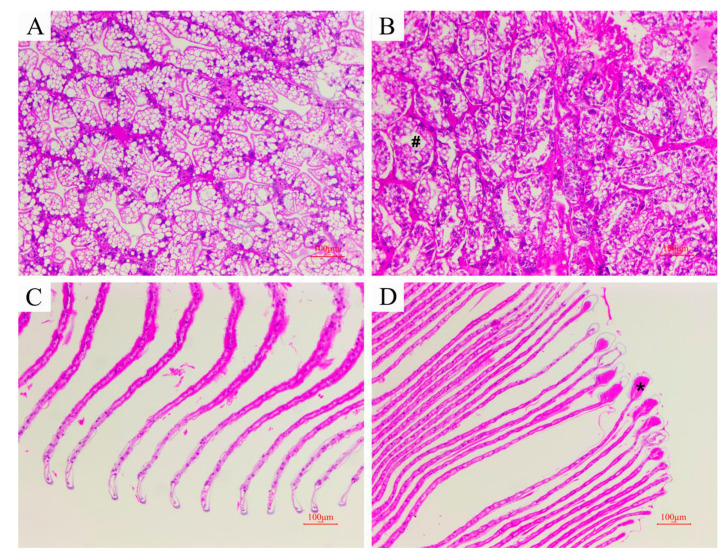
H&E-stained histological sections of *M. rosenbergii* (bar = 100 μm). (**A**) Hepatopancreas from the control group; (**B**) hepatopancreas from the infected group; (**C**) gill from the control group; (**D**) gill from the infected group. # shows loss of the star-like shape of the lumen; * shows clubbing at the tip of the gill filaments.

Histopathological examination revealed damage to the hepatopancreas and gills of *M. rosenbergii* infected with *A. veronii*. Noticeable alterations were observed in comparison to the control group. Specifically, the hepatic tubule lumen and the gap between hepatic tubules showed a significant enlargement. Additionally, the brush border, which is typically present, appeared to be absent in the infected prawns (Figure 1A,B). Furthermore, gill lesions were characterized by necrosis of the respiratory epithelial cells along with clubbing and degeneration observed at the distal end of the gill filaments (Figure 1C,D). 

### 3.3. Identification and Characterization of Isolate JDM1-1 

**Figure 2:**
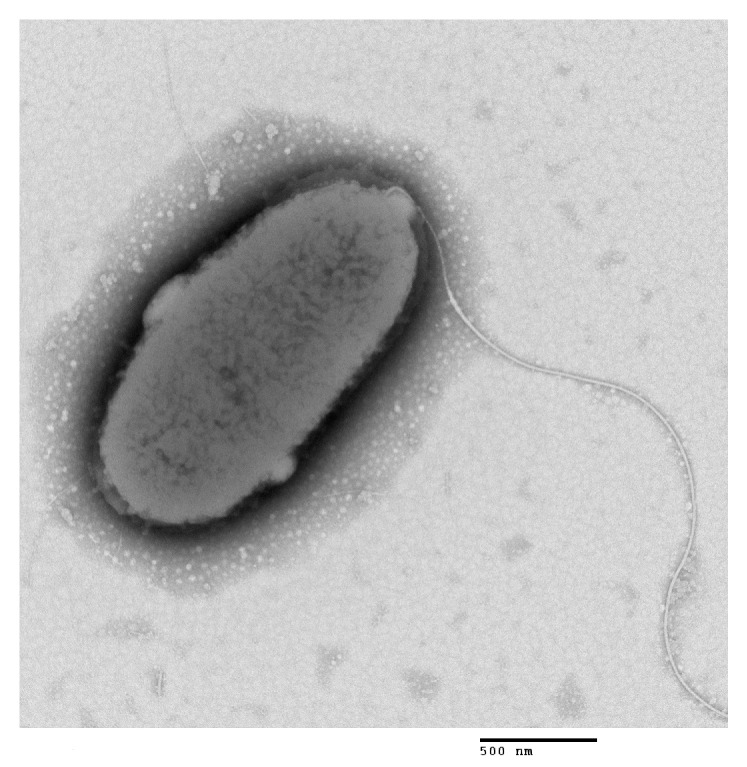
Electron micrograph of JDM1-1 showing single polar flagellum (bar = 500 nm).

The JDM1-1 isolate appeared gray-white in color and had a raised center, round shape, moist and smooth surface, and clear edges. Furthermore, TEM micrographs revealed that the JDM1-1 isolate was rod-shaped with round ends and was non-spore-forming. It had a width ranging from 1.0 to 1.2 μm and a length ranging from 2.0 to 2.2 μm. It achieved motility by a single polar flagellum (Figure 2).

The JDM1-1 isolate exhibited the following biochemical characteristics: positive for oxidase, motility, V-P test, salicin, maltose, mannose, mannitol, tartrate utilization, nitrate reduction, α-methyl-D-glucoside, and citrate utilization. On the other hand, JDM1-1 was negative for lactose, arabinose, sorbitol, inositol, H_2_S production, mucate utilization, and malonate utilization (Table 4). These characteristics are consistent with the description of *A. veronii* in Bergey’s Manual of Systematic Bacteriology.

**Figure 3:**
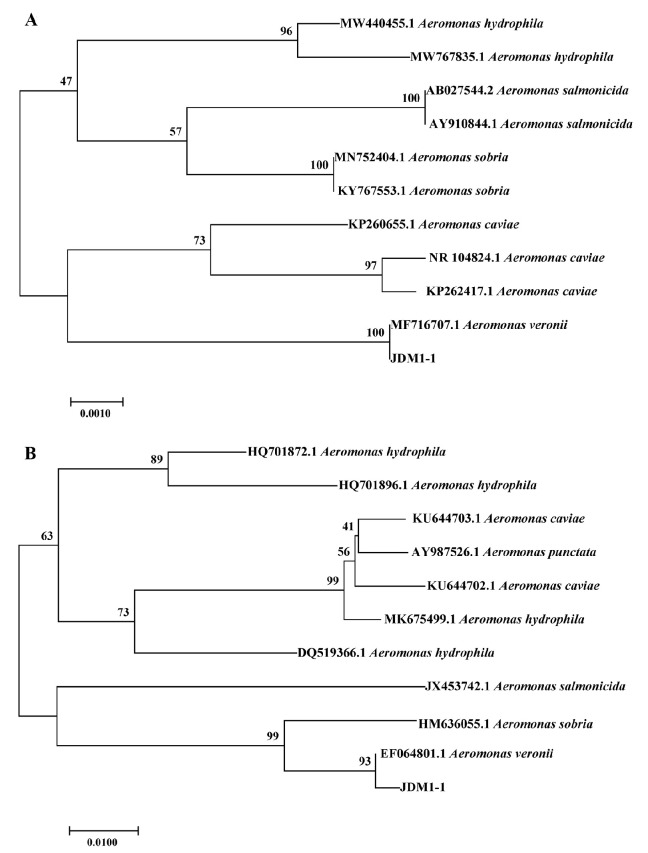
(**A**) Neighbor-joining (JDM1-1) phylogenetic tree based on partial 16S rRNA gene sequences. (**B**) Neighbor-joining (JDM1-1) phylogenetic tree based on partial gyrB gene sequences. Bootstrap values are shown beside the clades.

The 16S rRNA and *gyrB* genes from isolate JDM1-1 were sequenced and deposited in GenBank with accession numbers ON365878 and ON381308, respectively. The analysis of the 16S rRNA sequence revealed a 100% similarity to *A. veronii* strains (accession number: MF716707.1). Correspondingly, the *gyrB* sequence exhibited a 99% sequence homology to the *A. veronii* strain sequence (accession number: EF064801.1). In addition, the phylogenetic tree based on the partial 16S rRNA and *gyrB* gene sequences also showed JDM1-1 clustered with *A. veronii* (Figure 3).

### 3.4. Virulence Genes and Extracellular Products of Isolate JDM1-1

**Figure 4:**
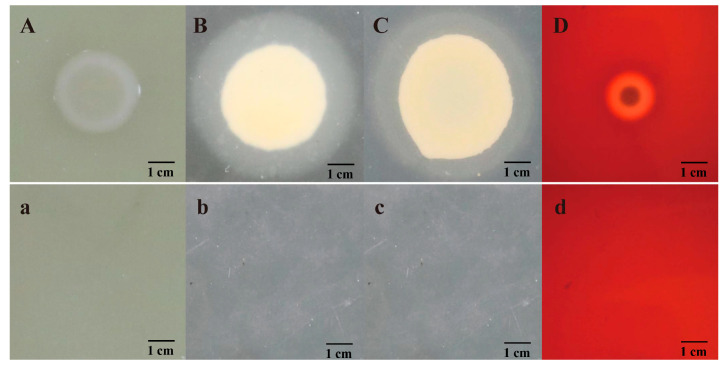
Determination of extracellular enzymes and hemolysin. (**a**,**A**): Hemolysin activities; (**a**): experimental group; (**A**): control group. (**b**,**B**): Gelatinase activities; (**b**): experimental group; (**B**): control group. (**c**,**C**): Lipase activities; (**c**): experimental group; (**C**): control group. (**d**,**D**): Lecithinase activities; (**d**): experimental group; (**D**): control group.

**Figure 5:**
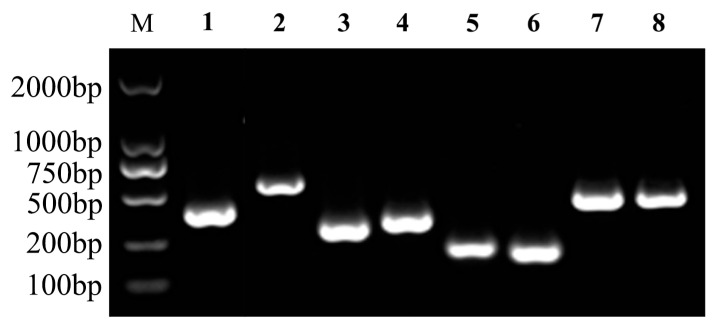
Agarose (1%) gel electrophoresis of PCR virulence gene products. M: 2000 bp DNA marker, Lane 1: *ompA*, 2: *lip*, 3: *hlyA*, 4: *flgA*, 5: *flgM*, 6: *flgN*, 7: *aer*, 8: *tapA*.

Extracellular product detection revealed that isolate JDM1-1 exhibited the production of lipase, lecithinase, gelatinase, and hemolysin (Figure 4). In addition, the presence of virulence-related genes, i.e., *aer*, *ompA*, *lip*, *tapA*, *hlyA*, *flgA*, *flgM,* and *flgN*, was observed in the isolate (Figure 5).

### 3.5. Expression Analysis of Immune-Related Genes of M. rosenbergii at Different Hours Post Infection

#### 3.5.1. Immune-Related Gene Expression in the Hepatopancreas after *A. veronii* Infection

**Figure 6:**
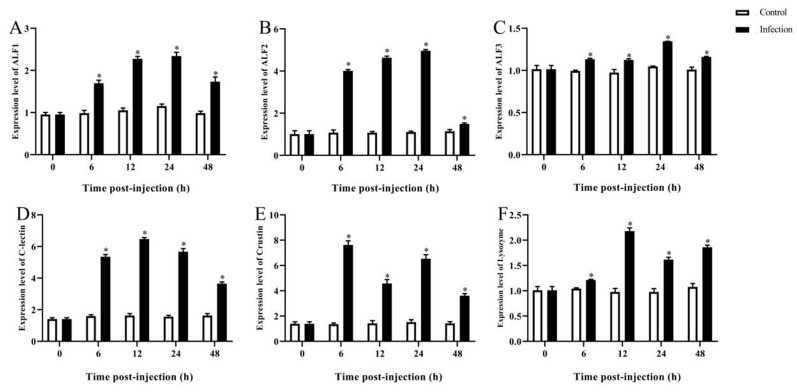
Immune-related gene expression in hepatopancreas after *A. veronii* infection. (**A**) *ALF1*, (**B**) *ALF2*, (**C**) *ALF3*, (**D**) *C-lectin*, (**E**) *Crustin*, (**F**) *lysozyme*. Data presented as mean ± SD, * *p* < 0.05.

Significant expression levels of *ALF1*, *ALF2*, and *ALF3* in the hepatopancreas of infected prawns were detected at 24 h post infection (hpi), with maximum fold changes of 2.46, 4.91, and 1.31 (*p* < 0.05), respectively. These expression levels were reduced from 24 to 48 h post infection (Figure 6A–C). The expression of *C-lectin* reached a peak with a fold change of 6.47 (*p* < 0.05) at 12 h, and then declined from 12 to 48 h (Figure 6D). Similarly, the *Lysozyme* gene showed a noticeable upregulation from 6 to 12 h, reaching its highest expression level with a fold change of 2.16 (*p* < 0.05) at 12 h (Figure 6F). As shown in Figure 6E, the expression of *Crustin* reached a peak at 6 hpi and was 7.5 times higher than the level in the control (*p* < 0.05).

#### 3.5.2. Immune-Related Gene Expression in the Intestines after *A. veronii* Infection

**Figure 7:**
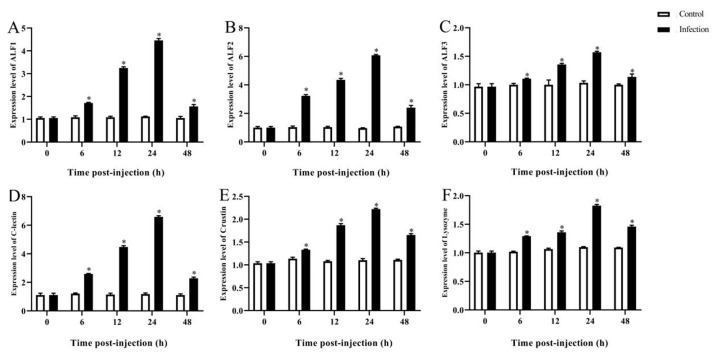
Immune-related gene expression in intestines after *A. veronii* infection. (**A**) *ALF1*, (**B**) *ALF2*, (**C**) *ALF3*, (**D**) *C-lectin*, (**E**) *Crustin*, (**F**) *lysozyme*. Data presented as mean ± SD, * *p* < 0.05.

As previously observed in the hepatopancreas, the different expression profiles of these genes in the intestines were analyzed and are shown in Figure 7. Increased expression levels of *ALF1*, *ALF2*, *ALF3*, *C-lectin*, *Lysozyme,* and *Crustin* were all observed during the 0 to 24 h period and reached 4.3-, 6.07-, 1.58-, 6.5-, 2.23- and 1.83-fold changes at 24 h (*p* < 0.05), respectively, followed by a drop at 48 hpi. The infected group maintained higher expression levels compared to the control group.

## 4. Discussion

Disease outbreaks caused by viral and bacterial pathogens have resulted in significant declines in the production of farmed crustaceans [23]. *M. rosenbergii*, an economically important cultured crustacean species, has experienced mass mortalities as a consequence of infections with various pathogens, including Taura Syndrome Virus (TSV), DIV1, *Vibrio alginolyticus*, *Vibrio cholerae*, *A. caviae,* and *A. veronii* [2]. In this study, strain JDM1-1 was identified as *A. veronii* based on the analysis of molecular information, including 16S rRNA and *gyrB* sequences, as well as biochemical reactions. Healthy prawns artificially infected with the isolated strain exhibited identical clinical symptoms to naturally deceased prawns. Additionally, *A. veronii* was successfully re-isolated from experimentally infected prawns. Moreover, the LD_50_ of strain JDM1-1 was 8.35 × 10^5^ CFU/mL, which was similar to the level found in *M. nipponense* [24]. Similarly, a previous study also showed that LD_50_ was 2.03 × 10^3^ cells/g for *A. veronii* to *M. rosenbergii* [12], which suggested that *A. veronii* is highly virulent to *M. rosenbergii*. Especially, the occurrence of mortality within 12 h after injection of JDM1-1 at concentrations of 1.8 × 10^8^ and 1.8 × 10^7^ CFU/mL indicated that this strain exhibits high pathogenicity, leading to a short incubation period; this result was similar to that achieved in a previous study in *Carassius auratus gibelio* [25]. As a result, it can be concluded that the mass mortality of *M. rosenbergii* was caused by *A. veronii* and *A. veronii* may be a potential threat to *M. rosenbergii* farms.

Previous research has established a correlation between the pathogenicity of bacteria and the existence of extracellular enzymes, which play crucial roles in the infection process. Specifically, in the genus *Aeromonas*, extracellular degradative enzymes such as lipase, lecithinase, and gelatinase have been identified and shown to contribute to virulence. These enzymes can act individually or in combination with other virulence factors, enhancing the pathogenic potential of the bacteria [26]. In this study, strain JDM1-1 exhibited the production of lipase, lecithinase, gelatinase, and hemolysin. Furthermore, virulence genes were recognized as significant factors that contributed to the pathogenicity of *A. veronii*. Aerolysin, coded by the *aer* gene, can damage intestinal epithelial tissue and is among the most significant and prevalent virulence factors in the infection of aquatic animals with *A. veronii* [27]. *OmpA* is essential in the adhesion of the *A. veronii* strain onto the surface of the intestinal tract, and its adhesion activity was markedly reduced in a gene knockout mutant [28]. Moreover, in previous studies, flagellar-mediated motility-related genes (*flgA*, *flgM,* and *flgN*) have been demonstrated to be crucial virulence factors in many bacterial species [29]. Hemolytic toxin-related gene (*hlyA*) is widely present in *Aeromonas* species and is involved in the species’ virulence [30]. In our study, we discovered that the isolated strain JDM1-1 possesses several virulence-related genes, including *aer*, *ompA*, *lip*, *tapA*, *hlyA*, *flgA*, *flgM,* and *flgN*, suggesting that the isolate has the potential to exhibit high pathogenicity.

Similar to other crustaceans, *M. rosenbergii* relies on innate immunity to defend itself against various pathogens. It is well known that various immune molecules of the innate immune system, including crustin, lectins, Toll-like receptors, and anti-lipopolysaccharide factors, have been studied [31,32]. In this study, we investigated the expression patterns of six immune-related genes in *M. rosenbergii* following infection with *A. veronii*. Antimicrobial peptides (AMPs) serve as essential components of the immune system by normalizing and destroying invading microbial pathogens. Among these AMPs, *crustin* has been confirmed as an important immune-related gene that can contribute to the innate defense system, safeguarding the organism against potential infections [33,34]. As another member of AMPs, anti-lipopolysaccharide factors (ALFs) could inhibit bacteria, viruses, and fungi by binding and neutralizing LPS [35]. In previous studies, *ALF* displayed significant antimicrobial properties against *Vibrio anguillarum*, *Vibrio penaeicida*, *Erwinia carotovora*, and *Staphylococcus aureus* and was able to protect against WSSV infection [36,37]. Lysozyme, known as muramidases zyme, is also a vital defense molecule in the innate immune system [38]. C-Type lectin (CTL), a pattern recognition receptor, plays an essential role in immune reactions such as bacterial and viral defense, phagocytosis, cell adhesion, prophenoloxidase activation, etc. [39,40]. In this study, increased expression levels of *ALF1*, *ALF2*, *C-Lectin*, *Crustin,* and *Lysozyme* were noticeable after *A. veronii* infection. Furthermore, even after their peak expression levels, the infected group consistently showed higher expression levels of these genes in comparison to the control group. This suggests that infection has a sustained impact on the transcriptional levels of these immune-related genes, indicating an ongoing immune response against the pathogen.

In conclusion, the present study identified JDM1-1 as *A. veronii* and as the bacterial pathogen causing mass mortality among *M. rosenbergii* by investigating its pathogenicity, virulence-related factors and genes. Furthermore, the expression levels of immune-related genes, including *ALF1*, *ALF2*, *C-Lectin*, *Crustin*, and *Lysozyme* changed significantly in response to *A. veronii* infection. This study revealed the pathogenic characteristics of *A. veronii* strain JDM1-1 and the host immune response, providing a theoretical basis for the prevention and control of *A. veronii* infection.

## Figures and Tables

**Table 1:** Sequences of primers for virulence gene detection.

Target Gene	Primer Sequence (5′–3′)	Product Size (bp)	Reference
*aer*	CCTATGGCCTGAGCGAGAAG	417	MH332385.1
	CCAGTTCCAGTCCCACCACT		
*ompA*	GCGGTTTATCGCTTTGGT	397	CP032839.1
	CACGCTTGGACTTGCTGA		
*flgA*	GGGACCTGCTGAGTGAAA	326	CP032839.1
	GACCGATACGGCACCTAC		
*flgM*	GCTACTGTCAAGCTGGACTC	194	CP032839.1
	AGATTGGCCTCGAAACTG		
*flgN*	AGTTGCTTGCTGCGATAGA	181	CP032839.1
	AGACGACGGTTTGAGACG		
*lip*	GTGCCGTCTGCCTTGGTGA	598	CP032839.1
	CCCGTCTATTGCGGGTTCGA		
*hlyA*	CGGACGATTATCAGGATGG	289	KY624579.1
	CAAGAACGAGTTTCAGTGGC		
*tapA*	ATGACCTCTAGCCCCAATA	550	CP050851.1
	ACCCGATTGATTTCTGCC		

**Table 2:** Sequences of primers for immune-related genes.

Target Gene	Primer Sequence (5′–3′)	Product Size (bp)
*ALF1*	GCCGATGGTGTCCTGGATG	157
	TCCATGCGTCGTCCTCCG	
*ALF2*	GGCACCAAACTCACTGGA	163
	CTTAGCACATGCGACCCTG	
*ALF3*	GAACTGCTGTCCAACCCTG	232
	CCGGATGCTCCTCCGTTATC	
*Crustin*	TGAAACTAACCTGTTCCAACG	165
	GAATGCCCTGCGATCCGAAGAA	
*C-lectin*	TGAAATTGCCTGTTGTTATG	180
	GGAGGGTGAGATGTAGCC	
*Lysozyme*	GACCTTGCGTCATGCCAGAT	182
	CCATGGGTTTATGTGCGTCTTC	
18S rRNA	TATACGCTAGTGGAGCTGGAA	182
	GGGGAGGTAGTGACGAAAAAT	

**Table 3:** Cumulative mortality for the determination of LD_50_ values in *M. rosenbergii* challenged with *A. veronii* by intramuscular injection at different concentrations.

Group	Infected Amount	Density (CFU/mL)	Dead Prawns after Challenge								Mortality
			12 h	1 d	2 d	3 d	4 d	5 d	6 d	7 d	
Test	30	1.8 × 10^8^	18	30	30	30	30	30	30	30	100.00%
	30	1.8 × 10^7^	7	13	19	25	27	28	29	29	96.67%
	30	1.8 × 10^6^	0	3	6	10	13	15	16	17	56.67%
	30	1.8 × 10^5^	0	0	2	3	5	5	5	6	16.67%
	30	1.8 × 10^4^	0	0	0	2	3	4	4	4	13.33%
Control	30	PBS	0	0	0	0	0	0	0	0	0

**Table 4:** Physiological and biochemical characteristics of isolate JDM1-1.

Biochemical Test	JDM1-1	*A.* * veronii* ^ a^	*A. sobria* ^ a^	*A. hydrophila* ^ a^
Oxidase	+	+	+	+
Motility	+	+	+	+
V-P test	+	+	d	+
O-F test	F	F	F	F
Lactose	-	-	-	d
Maltose	+	+	+	+
Mannitol	+	+	+	+
Mannose	+	+	+	[+]
Sorbitol	-	-	d	-
Inositol	-	-	-	-
Salicin	+	+	-	+
H_2_S production	-	-	-	+
α-methyl-D-glucoside	+	[+]	d	d
Tartrate utilization	+	[+]	d	-
Nitrate reduction	+	+	+	+
Citrate utilization	+	+	-	-
Mucate utilization	−	−	-	-
Malonate utilization	−	−	-	-

Note: “+”, positive; “−”, negative; “F”, fermentative; “[+]”, 76–89% of the strains are positive; “d”, 11~89%; “^a^”, data on *A. veronii*, *A. sobria,* and *A. hydrophila* from Bergey’s Manual of Systematic Bacteriology.

## Data Availability

Data are contained within the article.
